# Low-cost quasi-global optimization of expensive electromagnetic simulation models by inverse surrogates and response features

**DOI:** 10.1038/s41598-022-24250-1

**Published:** 2022-11-18

**Authors:** Slawomir Koziel, Anna Pietrenko-Dabrowska

**Affiliations:** 1 Engineering Optimization and Modeling Center, Reykjavik University, 102 Reykjavik, Iceland; 2 Faculty of Electronics, Telecommunications and Informatics, Gdansk University of Technology, 80-233 Gdansk, Poland

**Keywords:** Electrical and electronic engineering, Computational science

## Abstract

Conceptual design of contemporary high-frequency structures is typically followed by a careful tuning of their parameters, predominantly the geometry ones. The process aims at improving the relevant performance figures, and may be quite expensive. The reason is that conventional design methods, e.g., based on analytical or equivalent network models, often only yield rough initial designs. This is especially the case for miniaturized components featuring considerable electromagnetic (EM) cross couplings, or antenna systems with non-negligible radiator coupling (e.g., MIMO, closely-spaced arrays). For reliability reasons, parametric optimization is carried out using EM simulation tools, which is a time-consuming task. In many cases, designer needs to resort to a global search, especially when handling several objectives and constraints is necessary, or the high-frequency structure under design is overly complex. Combination of both aforementioned factors makes it no longer possible to rely on engineering insight, even to detect a promising region of the design space. Unfortunately, nature-inspired algorithms, commonly employed for solving these tasks typically exhibit significant computational expenditures. This paper proposes a simple yet efficient method for globalized search using a response feature approach and inverse regression surrogates. Owing to less nonlinear dependence of the feature point coordinates on the system variables (as compared to the original responses, e.g., *S*-parameter frequency characteristics), our methodology permits a rapid identification of the most appropriate regions of the parametric space, and further design tuning by means of local routines. At the same time, the overall optimization cost is comparable to the cost of local procedures. The proposed approach is validated using several high-frequency structures (a dual-band antenna, a microstrip coupler, an impedance matching transformer) optimized under different design scenarios. Global search capability and computational efficiency are demonstrated through comprehensive comparisons with multiple-start local search, as well as particle swarm optimizer, a representative nature-inspired algorithm.

## Introduction

Due to a growing complexity of contemporary high-frequency structures (antennas, microwave components, integrated photonic devices, etc.), carrying out a complete design cycle at the level of simplified representations such as analytical/semi-empirical formulas or equivalent networks is no longer feasible. Certain phenomena that exhibit non-negligible effects on the system performance, e.g. electromagnetic (EM) cross-coupling^1^, the presence of connectors^2^, housing^3^, mutual coupling of radiators^4^, can only be accounted for by means of a full-wave EM analysis. Appropriate capturing of these effects, along with the necessity of fulfilling stringent performance requirements (which includes realization of various functionalities such as tunability^5^, multi-band operation^6^, circular polarization^7^, but also maintaining small physical size of the devices^8–10^), leads to problems with obtaining reasonable initial designs or even locating the regions of the parameter space that contain such designs.

In this situation, parameter tuning becomes imperative. Nowadays, it is often performed using rigorous numerical optimization methods, as they are suitable for handling multiple objectives and constraints, as well as simultaneous adjustment of many parameters. Yet, several practical problems arise. EM-driven design, otherwise dictated by reliability, entails considerable computational costs, even in the case of local optimization. Further, the lack of good initial designs but also multimodality of the functional landscapes pertinent to many problems (e.g., pattern synthesis in antenna arrays^11^) call for global search, the cost of which may be considerably higher^12,13^.

The most widely used global optimization methods are by far population-based metaheuristics^14–20^. Well-known techniques of this sort include genetic^14^ and evolutionary algorithms^15^, particle swarm optimizers (PSO)^16^, differential evolution^17^, as well as numerous variations such as firefly algorithm^18^, harmony search^19^, grey wolf optimization^20^, and many others (e.g.,^21–26^). The underlying search mechanism is the exchange of information between a set of individuals (or agents) realized using appropriate exploratory (recombination) and exploitative (mutation) operators. This generally allows for identifying and exploiting the promising regions of the parameter space, in particular, escaping from the local optima. Metaheuristics are easy to implement and handle, but their global search capabilities come at a price of significant computational expenses, which may reach many thousands of objective function evaluations for a single algorithm run^20,23^. This is acceptable if the system evaluation cost is not of primary concern (e.g., for pattern synthesis of antenna arrays using analytical array factor models^20^), but turns prohibitive when directly optimizing full-wave EM models.

Population-based global search may still be a viable option if the EM simulation cost is reasonably low (e.g., a few seconds per analysis), or the available computational resources and licensing permit parallelization. Otherwise, more sophisticated approaches have to be employed, mostly involving surrogate modelling^27–29^. A representative example constitutes efficient global optimization (EGO) methods^30^, where a fast surrogate model (typically, kriging interpolation^31^ or Gaussian Process Regression^32^) is constructed along with conducting the search process itself. The infill samples are added based on various criteria that may aim at the parameter space exploration (i.e., improving the surrogate predictive power) or exploitation, i.e., finding the global optimum^33^. Other, yet related, options include application of machine learning techniques^34,35^, often in conjunction with sequential sampling methods^36^ as well as utilization of auxiliary surrogates to perform design space pre-screening^37,38^.

A construction of a globally accurate surrogate to replace expensive EM simulations altogether is a conceptually attractive alternative. In practice, due to the curse of dimensionality, it is limited to low-dimensional cases within restricted ranges of the system parameters^39,40^. This difficulty has been recently mitigated by the performance-driven modelling methods^41–43^, where the surrogate model domain is meticulously allocated to contain the designs being optimum with respect to the selected performance figures. This enables rendering reliable models over wide ranges of parameters and operating conditions^41,42^. Similar ideas were proposed in the context of multi-objective design, where initial identification of the Pareto front by means of single-objective optimization runs may permit one-shot surrogate construction at practically acceptable computational costs^44^. Such a model is subsequently used to yield initial approximations of the Pareto sets, further refined using local methods^44^. For this, but also other applications, additional speedup can be achieved by employing variable-fidelity EM simulations^45,46^.

The response feature approach, originally fostered for local optimization^47^, and later for surrogate modeling^48^, offers supplementary advantages. The optimization (or modeling) process is reformulated to be carried out at the level of appropriately defined characteristic points of the system outputs (e.g., frequency/level allocation of multi-band antenna resonances or local maxima of the in-band return loss response of a microwave filter), which is in opposition to handling the original responses, typically, frequency characteristics. The dependence of the feature point coordinates on the geometry parameters is normally much less nonlinear than for the standard outputs, which leads to flattening of the functional landscape to be handled. In the case of local optimization, it results in faster convergence^47^, whereas in the case of surrogate modeling it brings a considerable reduction of the number of training data samples required to render a reliable model^48^. This paper capitalizes on the response feature technology to realize globalized optimization of electromagnetic computational models. The major component is an inverse surrogate constructed at the level of feature points extracted from a set of random observables. The predictions generated by the surrogate allow for rapid identification of the promising regions of the parameter space and yielding a reasonable initial design, which only needs to be tuned in a local sense. At the same time, the computational complexity of the proposed procedure is comparable to that of local procedures. Our methodology is validated using several high-frequency structures (a dual-band antenna, a microstrip coupler, an impedance matching transformer), optimized under different scenarios. The global search capability and computational efficiency are demonstrated through comprehensive comparisons with multiple-start local search, as well as particle swarm optimizer (PSO), a representative nature-inspired population-based procedure.

*Methods*. The proposed optimization approach has been developed in accordance with the methodology itemized below:

1. Conceptual development of the algorithmic framework for globalized optimization of high-frequency structures;
2. Computer implementation of the algorithm and debugging;
3. Preparing a library of test problems including their computational (EM-simulation) models;
4. Setting up control parameters of the optimization framework;
5. Implementation of benchmark algorithms;
6. Conducting numerical experiments;
7. Comparative analysis of the results;
8. Extraction of the final conclusions and findings.

The proposed optimization framework has been implemented in MATLAB. Whereas the EM models of all the high-frequency components used in this work as verification case studies are evaluated using time-domain solver of CST Microwave Studio. All the simulations have been performed on Intel Xeon 2.1 GHz dual-core CPU with 128 GB RAM.

## Globalized search using inverse regression surrogates and response features

This section introduces a globalized search procedure proposed in this work. We begin by discussing its two fundamental components: a response feature approach employed to flatten the functional landscape of the objective function pertinent to the design problem at hand, as well as the inverse regression model utilized to facilitate identification of the promising regions of the parameter space. Both mechanisms are critical to enable global search capability at low computational cost, comparable to that of local optimizers as demonstrated in “Demonstration case studies” section. Another important ingredient of the procedure is a local optimization routine outlined in “Design refinement by local search” section, which permits carrying out fine-tuning of the system parameters. The operation of the entire optimization framework is discussed in “Optimization framework” section.

### Response features for global search

The need for global search emerges for many high-frequency structures, either due to the lack of a reasonable initial design, or a multimodality (the presence of multiple local optima) of the functional landscape pertinent to the cost function quantifying the design utility. The primary challenge of global optimization is the necessity of carrying of the search process within the entire parameter space, which is often highly dimensional. Furthermore, the responses of electromagnetic computational models exhibit significant variability, e.g., considerable shifts of operating frequencies across the design space. For example, conducting the search process using nature-inspired algorithm, otherwise suitable for such purposes, entails considerable computational expenses, especially when executed at the level of full-wave simulation models.

**Figure 1:**
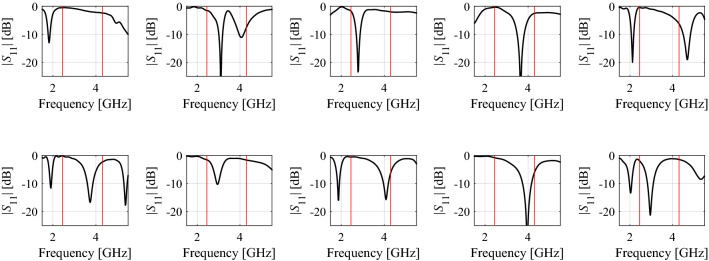
Exemplary reflection responses of a dual-band antenna along with the target operating frequencies marked using vertical lines (here, 2.45 GHz and 4.3 GHz). Launching the local search aimed at, e.g., reduction of the antenna reflection in the vicinity of the target frequencies (and formulated in a minimax sense), from the majority of the presented designs would likely not bring satisfactory results as a direct consequence of a deficient initial allocation of the reflection characteristic minima.

**Figure 2:**
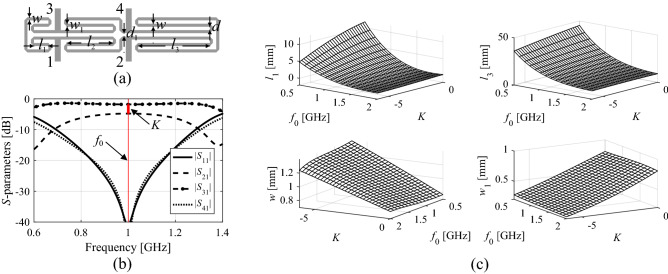
Miniaturized rat-race coupler^53^: (**a**) circuit geometry, (**b**) typical *S*-parameter response, (**c**) two-dimensional surfaces corresponding to (approximately) optimum designs obtained for various target operating frequencies and power split ratios, here, shown for the selected geometry parameters.

Figure 1 shows example reflection responses of a dual band antenna at various points of the parameter space. It is clear that using these particular designs as starting points for local optimization may lead to a failure of the optimization process, e.g., because the initial allocation of antenna resonances is incompatible with the target ones, or the resonances are not clearly distinguished. On the other hand, following the underlying physical properties of passive components, the parameters of high-frequency structures corresponding to designs that are optimum with respect to particular performance figures are largely correlated^49–51^. For example, dimension scaling of antenna or microwave components with respect to the operating frequency (or frequencies in the case of multi-band structures), substrate parameters, power split ratio (in the case of couplers), etc., requires synchronized adjustment of the design variables. In other words, the optimum designs are normally allocated along low-dimensional manifolds (surfaces) of the dimensionality equal to the number of operating conditions or performance figures considered for a given design task^52^. This observation has led to the development of a paradigm of constrained modeling, in which the modeling process is restricted to this part of the design space, where designs of high-quality reside^52^. As the number of operating conditions is normally considerably lower than the number of parameters of the system at hand, constrained modelling effectively reduces the modelling task dimensionality^52^. These manifolds are usually regular as indicated in Fig. 2, using the example of a miniaturized rat-race coupler^53^.

Capturing the correlations between the parameter values corresponding to optimum designs is a key to accelerate the global optimization process according to the methodology proposed in this paper. Typically, to assess a particular design in relation to the optimum design manifold, it is sufficient to consider only particular portions of the system outputs, here, referred to as response features^47^. The feature points are to be extracted from the complete characteristics, and they determine the relations between these and the design objectives. For example, if a goal is to optimize a dual-band antenna so that its resonances are allocated at given target frequencies and, at the same time, reflection levels therein are to be minimized, appropriate response features may simply be the frequency and level coordinates of the resonances. If, however, we aim at maximization of a fractional bandwidth around the target frequencies, then the frequencies corresponding to − 10 dB reflection level would be more suitable. For other structures, the choice of the feature points may be more involved (see, e.g.,^54,55^).

It should be emphasized that a rigorous mathematical definition of response features has not been formulated. This is because they are very much problem dependent, as the examples of the previous paragraph indicate. Thus, there exist no definition, which would be sufficiently general to cover all possible cases that may emerge when solving practical design tasks. This is a limitation of the response feature technology. A generalized definition of the response features has been given in^56^ for antenna input characteristics. Despite covering merely one type of antenna response, the definition of^56^ is intricate, which confirms that providing a rigorous mathematical definition of response feature for all types of characteristics of antenna and microwave structures that may be of interest in high-frequency design is far from trivial. Therefore, as explained above, the response features need to be defined in relation to a specific design task.

**Figure 3:**
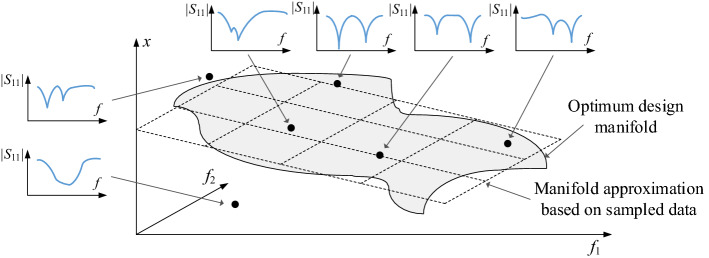
Conceptual illustration of the use of response features for identification of the promising regions of the parameter space. Shown are: optimum design manifold (here, demonstrated for a dual-band antenna optimized with respect to two operating frequencies *f*_1_ and *f*_2_), the vertical axis being one of antenna geometry parameters; black circles corresponding to random observables with their response features (here, antenna resonance allocation) extracted. Note that the observables are not optimized, therefore, they are generally away from the optimum design manifold; however, they can still be used to produce a rough approximation of the manifold by associating the response features with the objective space of *f*_1_ and *f*_2_ (cf. “Identification of promising regions by inverse regression models” section).

Qualitatively, given a number of random designs (observables) and their response features extracted, one may attempt to use this information to at least roughly approximate the optimum design manifold. The model obtained this way can be employed as a predictor rendering a point in a reasonable proximity of the optimum design, corresponding to the target values of the performance figures relevant to the design problem at hand. A graphical illustration of this concept has been shown in Fig. 3. Its quantification will be discussed at length in “Identification of promising regions by inverse regression models” section.

### Identification of promising regions by inverse regression models

As indicated in “Response features for global search” section, the response features can be effectively employed to identify the promising regions of the parameter space, i.e., the vicinity of the optimum design manifold. This section provides quantification of this concept by means of inverse regression surrogates. We start by introducing the necessary notation. Let ***R***(***x***) denote the response (e.g., *S*-parameters vs. frequency) of the EM-simulation model of the structure at hand, where ***x*** is a vector of designable variables.

The task is to find

1 $${\varvec{x}}^{*} = {\varvec{x}}^{*} ({\varvec{F}}) \in \arg \mathop {\min }\limits_{{{\varvec{x}} \in X}} U({\varvec{x}},{\varvec{F}})$$

where *U*(***x***,***F***) is a scalar merit function to be minimized, ***F*** = [*F*_1_ … *F*_*N*_]^*T*^ is a target vector of design objectives (hereafter it will be referred to as the target objective vector), whereas *X* denotes the parameter space usually delimited by lower and upper bounds on design variables. Two examples follow. If the goal is to minimize the reflection coefficient at the intended operating frequencies *F*_*k*_ of a multi-band antenna, the merit function could be defined in a minimax sense as

2 $$U({\varvec{x}},{\varvec{F}}) = \mathop {\max }\limits_{{}} \left\{ {|S_{11} ({\varvec{x}},F_{1} )|, \ldots ,|S_{11} ({\varvec{x}},F_{N} )|} \right\}$$

If the goal is to design a coupler for a particular power split ratio *K* =|*S*_21_(***x***,***F***)| −|*S*_31_(***x***,***F***)| at the operating frequency *f*_0_, then one may define *F*_1_ = *f*_0_, *F*_2_ = *K* and

3 $$U({\varvec{x}},{\varvec{F}}) = \mathop {\max }\limits_{{}} \left\{ {|S_{11} ({\varvec{x}},F_{1} )|,|S_{41} ({\varvec{x}},F_{1} )|} \right\} + \beta \left( {F_{2} - (|S_{21} ({\varvec{x}},F_{1} )| - |S_{31} ({\varvec{x}},F_{1} )|} \right)^{2}$$

Here, the primary objective is the minimization of the reflection and isolation at the operating frequency. The required power split (*F*_2_ = *K*) is enforced by adding a regularization factor (second term in (3)) with *β* being a penalty coefficient.

The optimum design manifold *O*(*F*) is the set of points of the form x^*^(***F***) for all ***F*** ∈ *F*, where *F* is the objective space pertinent to the design task (e.g., determined by the lower and upper bounds for the objectives *F*_*k*_). We also denote as F^*^ a particular target objective vector being a target for the specific design task considered for the structure at hand.

Let ***p***(***x***) = [*p*_1_(***x***) … *p*_*L*_(***x***)]^*T*^ be the vector of response features extracted from the simulated characteristics of the structure under design at ***x***. Further, let *s*_*k*_(***p***(***x***)), *k* = 1, …, *N*_*T*_, be the scalar functions mapping the features into the space *F*_*T*_ of auxiliary objective vectors ***F***_*T*_ ∈ *F*_*T*_. Generally, *F*_*T*_ coincides with the objective space *F* (in particular, *N*_*T*_ = *N*); however, in some instances, they may be different (cf. Section “Demonstration case studies)” section, for example, when the assessment of the design utility (for the purpose of yielding the initial design for further refinement) requires a larger number of scalar quantifiers than those contained in the target objective vectors ***F***.

The response features, aggregated in the vector ***p***, are defined for specific circuit characteristics based on their shape and by taking into account the design objectives (operating parameters). The reason for employing the function *s*_*k*_(***p***(***x***)) is that the response features and the design objectives may be in some situations identical, yet, this is not always the case. For example, if the design objective is to allocate the antenna resonance at a required frequency *f*_0_, then the design objective *F* and the response feature *f*_0_ are identical, i.e., we have *F* = *f*_0_. Still, in many cases, the relationship between the response features and the design objectives is not so straightforward. Consider the microwave coupler, which is to operate at a target center frequency *f*_0_. For this structure, the response features are defined as the minima of *S*_11_(***x***,***F***) and *S*_41_(***x***,***F***) characteristics, and they need to be controlled by the optimization process. We have ***p*** = [*f*_0.*S*11_ *f*_0.*S*41_]^*T*^, where *f*_0.*S*11_ and *f*_0.*S*41_ are the frequencies corresponding to minima of the reflection and isolation characteristics. However, the relevant design objective in this case is the circuit operating frequency *f*_0_, i.e., *F* = *f*_0_. Therefore, a function *s*(***p***) allows us to map the space of the response features into the objective space if necessary. In this case it might be defined as *s*_1_(***p***(***x***)) = (*f*_0.*S*11_ + *f*_0.*S*41_)/2, i.e., the operating frequency of the circuit is estimated as the average of the frequencies of the said minima.

The functions *s*_*k*_ are defined by the designer and determine the approximate allocation of the design ***x*** in terms of its performance figure values in relation to x^*^(***F***). We also denote ***s***(***p***) = [*s*_1_(***p***) … *s*_*NT*_(***p***)]^*T*^. For simplicity of notation, we omit the dependence of the vector of features ***p*** on the parameter vector ***x***. Further, let ***F***_*T*_ = [*F*_*T.*1_ … *F*_*T.NT*_]^*T*^ be a target (auxiliary) objective vector, also user-defined, determining the design quality in terms of response features. The corresponding merit function *E*(***p***) quantifying the design quality in the above sense is *E*(***p***) =||***s***(***p***) − ***F***_*T*_||^2^. We will denote by ***F***_T_^*^ the particular auxiliary vector corresponding to the target objective vector F^*^ (pertinent to the specific design task).

Going back to the example of a multi-band antenna considered before and the merit function (2), the appropriate choice of the response features would be ***p*** = [*f*_0.1_ … *f*_0.*N*_* l*_1_ … *l*_*N*_]^*T*^, where *f*_0.*k*_ and *l*_*k*_ are the frequency and the |*S*_11_(***x***,***F***)|-value at the *k*th antenna resonance. In this case, one may define *s*_*k*_(***p***) = *f*_0.*k*_ if the assessment of the allocation of a particular design is to be based on the distance between the actual and the target operating frequencies. Then, the vector ***F***_*T*_ coincides with the target objective vector ***F*** (in particular, *N*_*T*_ = *N*), and *E*(***p***) =||[*f*_0.1_ … *f*_0.*N*_]^*T*^ − ***F***||^2^.

In the case of the coupler (cf. (3)), the response feature vector may be defined as ***p*** = [*f*_0.*S*11_ *f*_0.*S*41_ *l*_*S*11_ *l*_*S*21_ *l*_*S*31_ *l*_*S*41_]^*T*^, where *f*_0.*S*11_ and *f*_0.*S*41_ are the frequencies corresponding to minima of the reflection and isolation characteristics with *l*_*S*11_ and *l*_*S*41_ being the corresponding levels, whereas *l*_*S*21_ and *l*_*S*31_ are the levels of the transmission characteristics at the frequency (*f*_0.*S*11_ + *f*_0.*S*41_)/2. Here, the functions *s*_*k*_ would be defined as *s*_1_(***p***) = (*f*_0.*S*11_ + *f*_0.*S*41_)/2, and *s*_2_(***p***) = *l*_*S*21_ − *l*_*S*31_. Again, the vector ***F***_*T*_ coincides with ***F*** and *E*(***p***) =||[(*f*_0.*S*11_ + *f*_0.*S*41_)/2 *l*_*S*21_ − *l*_*S*31_]^*T*^ − ***F***||^2^. As mentioned before, although in general, *N*_*T*_ = *N* and ***F***_*T*_ = ***F***, this may not be the case for specific situations (cf. Section "Demonstration case studies").

The aforementioned concepts will be employed to identify the promising regions of the parameter space *X*, and delimited using the lower and upper bounds ***l*** and ***u***, respectively. Let ***x***_*r*_^(*j*)^ = [*x*_*r*.1_^(*j*)^ … *x*_*r*.*n*_^(*j*)^]^*T*^, *j* = 1, …, *N*_1_, be the set of random points allocated (preferably uniformly) in *X*. With each point, we associate the corresponding feature vector ***p***(***x***_*r*_^(*j*)^) and *E*^(*j*)^ = *E*(***p***(***x***_*r*_^(*j*)^)). Here, the dependence of the response feature on the particular random observable vector ***x***_*r*_^(*j*)^ has been explicitly given to indicate that the value of the merit function *E*^(*j*)^ corresponds to the particular vector ***x***_*r*_^(*j*)^; in the following, for simplicity of notation, this dependence will be omitted and we will simply use *E*^(*j*)^. If some of the pre-defined feature points do not exist (e.g., one or more of the multi-band antenna resonances is not clearly distinguished) the corresponding sample is removed from the set. Without loss of generality, we assume that the observables ***x***_*r*_^(*j*)^ are ordered so that *E*^(1)^ ≤ *E*^(2)^ ≤ … ≤ *E*^(*N*1)^. In particular, the first sample point represents the design that is the closest to the auxiliary objective vector ***F***_*T*_ in the sense described in the previous paragraphs.

In the next step, we use *N*_2_ ≤ *N*_1_ of the best sample points to establish an inverse regression surrogate ***L***(***s***(***p***)) defined over *F*_*T*_ with the values in *X*, which will be used to yield an approximation of the design that is as close as possible to the target one, i.e., the design that minimizes *E*(***p***). Here, the surrogate is assumed to be a linear function of ***s*** due to the fact that *N*_1_ is generally small, and practical allocation of the observables may be far from uniform. Under these circumstances, linear approximation ensures better extrapolation capabilities than interpolative models (e.g., kriging^31^). Thus, we have

4 $${\varvec{L}}({\varvec{s}}({\varvec{p}})) = \left[ {\begin{array}{*{20}c} {l_{0.1} + \sum\nolimits_{i = 1}^{{N_{T} }} {l_{i.1} s_{k} ({\varvec{p}})} } \\ \vdots \\ {l_{0.n} + \sum\nolimits_{i = 1}^{{N_{T} }} {l_{i.n} s_{k} ({\varvec{p}})} } \\ \end{array} } \right]$$

The model coefficients are obtained by solving the regression problems

5 $${\varvec{L}}({\varvec{s}}({\varvec{p}}({\varvec{x}}_{r}^{(j)} )) = {\varvec{x}}_{r}^{(j)} ,\quad j = { 1}, \ldots ,N_{{2}}$$

where the dependence of the regression surrogate ***L***(***s***(***p***(***x***_*r*_^(*j*)^))) on the specific random observable vector ***x***_*r*_^(*j*)^ has been explicitly shown. In other words, the affine plane corresponding to the surrogate model output provides the best possible approximation of the observable set in the least square sense. In practice, we intend to put more emphasis on the high-quality samples, i.e., those characterized by small values of *E*^(*j*)^. Toward this end, let us define the weights *w*_*j*_ = 1/*E*^(*j*)^ and the weight matrix ***W*** = diag(*w*_1_,…,*w*_*N*2_). The original regression problem (5) is then replaced by the weighted one of the form

6 $${\varvec{A}}^{T} {\varvec{WAl}}_{{\varvec{L}}} = {\varvec{A}}^{T} {\varvec{WX}}$$

where

7 $${\varvec{l}}_{{\varvec{L}}} = \left[ {\begin{array}{*{20}c} {l_{0.1} } & {l_{1.1} } & \cdots & {l_{{N_{T} .1}} } \\ \vdots & {} & \ddots & \vdots \\ {l_{0.n} } & {l_{1.n} } & \cdots & {l_{{N_{T} .n}} } \\ \end{array} } \right]^{T}$$

8 $${\varvec{A}} = \left[ {\begin{array}{*{20}c} 1 & {s_{1} ({\varvec{p}}({\varvec{x}}_{r}^{(1)} ))} & \cdots & {s_{{N_{T} }} ({\varvec{p}}({\varvec{x}}_{r}^{(1)} ))} \\ \vdots & {} & \ddots & \vdots \\ 1 & {s_{1} ({\varvec{p}}({\varvec{x}}_{r}^{{(N_{2} )}} ))} & \cdots & {s_{{N_{T} }} ({\varvec{p}}({\varvec{x}}_{r}^{{(N_{2} )}} ))} \\ \end{array} } \right]$$

and

9 $${\varvec{X}} = \left[ \begin{gathered} {\varvec{x}}_{r}^{(1)T} \\ \vdots \\ {\varvec{x}}_{r}^{{(N_{2} )T}} \\ \end{gathered} \right]$$

The least-square solution to (6) is given as

10 $${\varvec{l}}_{{\varvec{L}}} = \left( {{\varvec{A}}^{T} {\varvec{WA}}} \right)^{ - 1} {\varvec{A}}^{T} {\varvec{WX}}$$

The inverse surrogate can be used to produce the design ***x***_T_^*^ that provides the best approximation of the target objective vector ***F***_T_^*^ as follows

11 $${\varvec{x}}_{T}^{*} = {\varvec{L}}\left( {\left[ {1\;\;({\varvec{F}}_{T}^{*} )^{T} } \right]^{T} } \right) = {\varvec{l}}_{{\varvec{L}}}^{T} \left[ {1\;\;({\varvec{F}}_{T}^{*} )^{T} } \right]^{T}$$

Provided this design is of sufficient quality, the vector ***x***_T_^*^ may be used as a starting point for local optimization. Otherwise, it might be further refined by supplementing the existing observable set with the infill samples, rebuilding the inverse model, and making another prediction according to (11). “Optimization framework” section provides the details of the optimization framework that implements the aforementioned iterative procedure.

The fundamental advantage of constructing the feature-based inverse surrogate is only somewhat nonlinear dependence between the feature point coordinates and the geometry parameters of the structure under design (cf. Fig. 2). Owing to this, even a relatively small number of observables ***x***_*r*_^(*j*)^ may be sufficient to identify a promising region of the parameter space, i.e., containing the optimum design x^*^(F^*^).

### Design refinement by local search

Local optimization is performed using the trust-region (TR) gradient search algorithm with numerical derivatives^57^. The algorithm produces a generates a sequence ***x***^(*i*)^, *i* = 0, 1, …, of approximations to x^*^ as

12 $${\varvec{x}}^{(i + 1)} = \arg \mathop {\min }\limits_{{{\varvec{x}};\; - {\varvec{d}}^{(i)} \le {\varvec{x}} - {\varvec{x}}^{(i)} \le {\varvec{d}}^{(i)} }} U({\varvec{G}}_{{}}^{(i)} ({\varvec{x}}),{\varvec{F}}^{*} )$$

The model ***G***^(*i*)^(***x***) locally approximates ***R***(***x***) at ***x***^(*i*)^; here, we employ a first-order Taylor expansion given as

13 $${\varvec{G}}^{\left( i \right)} \left( {\varvec{x}} \right) \, = {\varvec{R}}\left( {{\varvec{x}}^{(i)} } \right) + {\varvec{J}}_{{\varvec{R}}} \left( {{\varvec{x}}^{(i)} } \right) \cdot \left( {{\varvec{x}} - {\varvec{x}}^{(i)} } \right)$$

In (13), the Jacobian ***J***_***R***_(***x***) estimatation requires finite differentiation. The trust region is delimited by ***x***^(*i*)^ − ***d***^(*i*)^ ≤ ***x*** ≤ ***x***^(*i*)^ + ***d***^(*i*)^ (i.e., its lower and upper bounds), with the inequalities interpreted component-wise. The size ***d***^(*i*)^ of the search region is established with conformance to the standard TR algorithm setup^57^. The adopted definition of the trust region permits handling variables featuring considerably different ranges, as ***d***^(0)^ (i.e., the size of the initial TR region) becomes proportional to the parameter space size ***u*** − ***l***), thus eliminating the necessity of scaling the antenna parameters.

Upon each successful iteration, the TR algorithm (12) adjusts the local model (i.e., re-evaluates the Jacobian), hence, its cost equals *n* + 1 EM simulation of the component under design. The local search process may be accelerated using the expedited versions of the TR algorithm involving sparse Jacobian updates, as described in^58^ or^59^. Here, after the first iteration, we simply replace finite differentiation by a rank-one Broyden formula^60^, which is justified by the availability of good-quality initial design found by the global search stage.

### Optimization framework

This section describes the entire optimization framework involving the components discussed in “Response features for global search” through “Design refinement by local search” sections. The control parameters of the algorithm (some of which have been already mentioned before) include:

- *N*_1_—the number of initial random samples ***x***_*r*_^(*j*)^, here, using LHS^61^ ﻿﻿(cf. “Identification of promising regions by inverse regression models” section);
- *N*_2_—the number of best samples selected to construct the inverse surrogate;
- *N*_3_—the number of infill samples added to the sample pool per iteration;
- *N*_*max_global*_—maximum number of EM evaluations for the global search stage;
- *N*_*max_local*_—maximum number of EM evaluations for the local search state;
- ***x***_*best*_—best design found so far in the process;
- *U*_*local*_—threshold value for *U*(***x***_*best*_,F^*^) to enter the local search stage;
- *E*_*reduce*_—threshold value for *E*(***p***(***x***_*best*_)) to reduce the sampling region (infill sampling);
- *a*_*red*_—multiplication factor for sampling region reduction.

**Figure 4:**
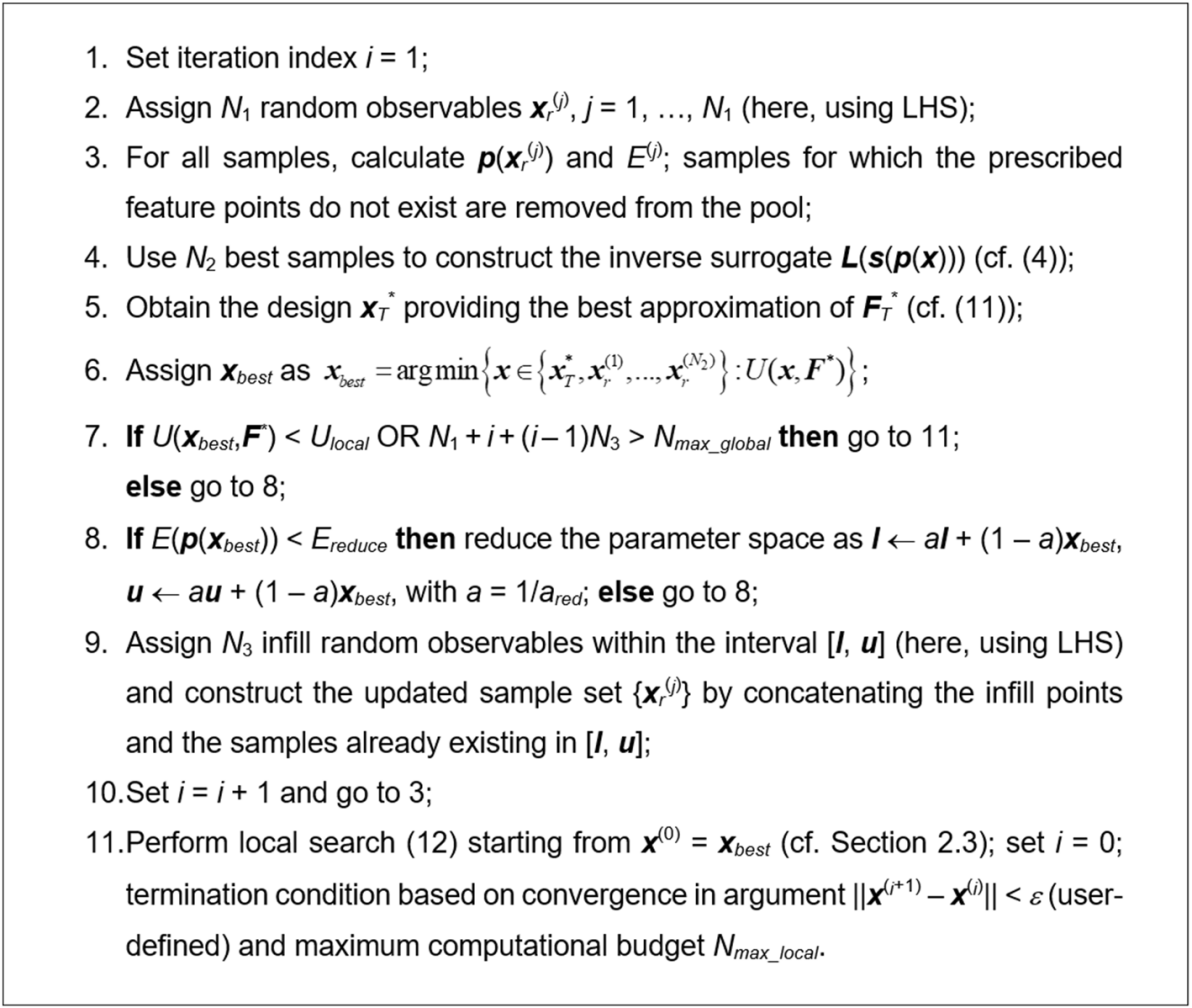
The workflow of the proposed procedure for quasi-global optimization of high-frequency structures.

The operation of the algorithm is shown in Fig. 4. In Fig. 4, Steps 3 through 10 pertain to the global search step, whereas Step 11 corresponds to the local refinement. Similarly as in  “Identification of promising regions by inverse regression models” section, F^*^ and ***F***_T_^*^ denote the target objective and auxiliary objective vectors; ***l*** and ***u*** are lower and upper bounds determining the parameter space.

**Figure 5:**
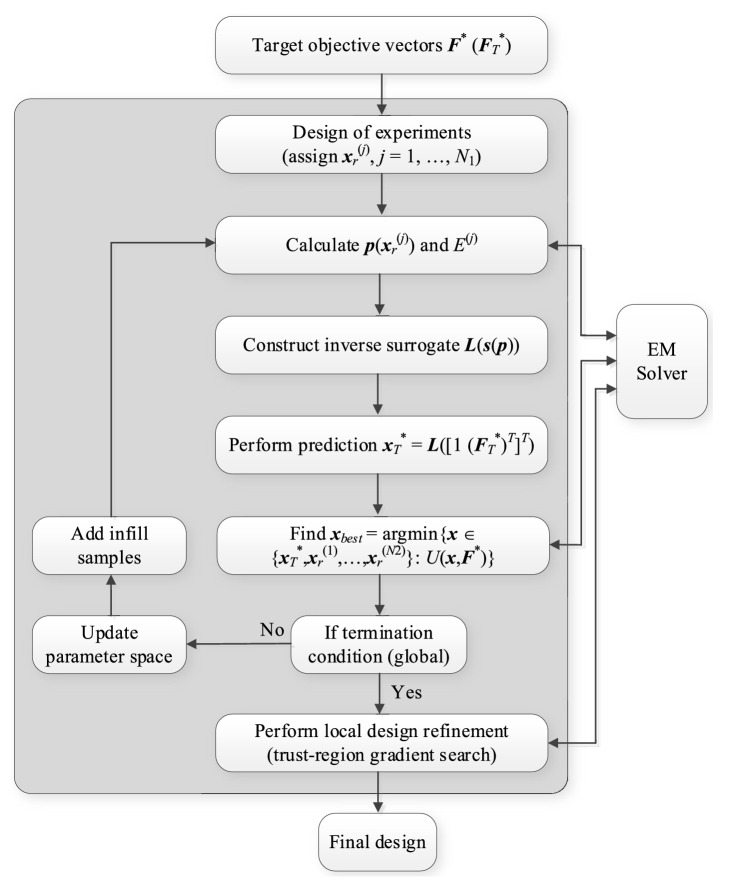
Flow diagram of the proposed globalized optimization framework involving response features and inverse surrogates.

Note that in the above algorithm, global search is followed by the local refinement, which concludes the process. In principle, the procedure can be iterated; however, in order to demonstrate the benefits of involving the response features into the process, for all demonstration examples considered in “Demonstration case studies” section, only one pass is executed. The number *N*_1_ + *i* + (*i* − 1)*N*_3_ in Step 7 is the overall number of EM simulations executed at this stage of the process, including: (i) the initial sampling, (ii) system evaluation at the surrogate-predicted design ***x***_T_^*^, and (iii) the infill sampling. If, upon removing samples for which the prescribed feature points do not exist, the remaining number of samples is smaller than *N*_2_, the surrogate is constructed if possible, otherwise (i.e., if the number of samples is smaller than *N*_*T*_ + 1), the surrogate is not constructed in a given iteration and ${\mathbf{x}}_{best} = \arg \mathop {\min }\limits_{{}} \left\{ {{\mathbf{x}} \in \left\{ {{\mathbf{x}}_{r}^{(1)} , \ldots ,{\mathbf{x}}_{r}^{{(N_{2} )}} } \right\}{:}\,U({\mathbf{x}},{\mathbf{F}}^{*} )} \right\}$ A graphical illustration of the optimization process has been shown in Fig. 5 in the form of a flow diagram.

## Demonstration case studies

This section demonstrates the operation and performance of the proposed optimization technique using three high-frequency structures: a dual-band dipole antenna, a miniaturized rat-race coupler, and a two-section compact impedance matching transformer. In all cases, the results obtained using the algorithm of “Globalized search using inverse regression surrogates and response features” section have been compared to a representative nature-inspired method (here, particle swarm optimizer, PSO^62^), in terms of computational cost and design quality. Furthermore, the verification structures have been optimized using gradient-based search executed from random starting points. Less-than-perfect success rate of the latter indicates that globalized optimization is indeed required for the considered design tasks, except for the last structure.

### Verification case studies

**Figure 6:**
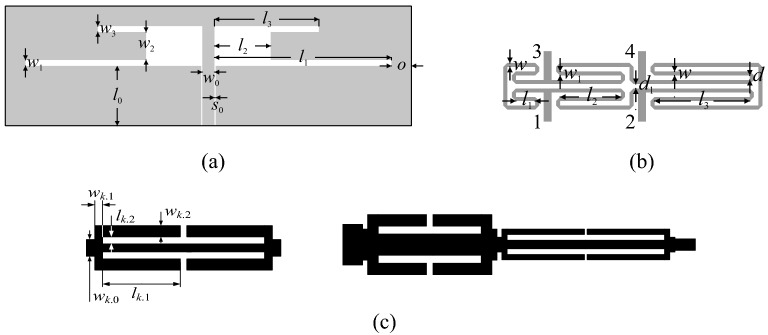
Verification structures: (**a**) uniplanar dual-band dipole antenna^63^, (**b**) miniaturized rat-race coupler (RRC)^64^, (**c**) compact two-section impedance matching transformer: CMRC cell (left) and complete transformer structure (right)^65^; *k* refers to a section index.

Figure 6 shows the verification devices utilized to demonstrate and validate the proposed optimization approach. These include:

- A uniplanar dual-band dipole antenna shown in Fig. 6a (Structure I)^63^;
- A miniaturized rat-race coupler (RRC) shown in Fig. 6b (Structure II)^64^;
- A compact two-section CMRC-based impedance matching transformer shown in Fig. 6c (Structure III)^65^.

**Figure 7:**
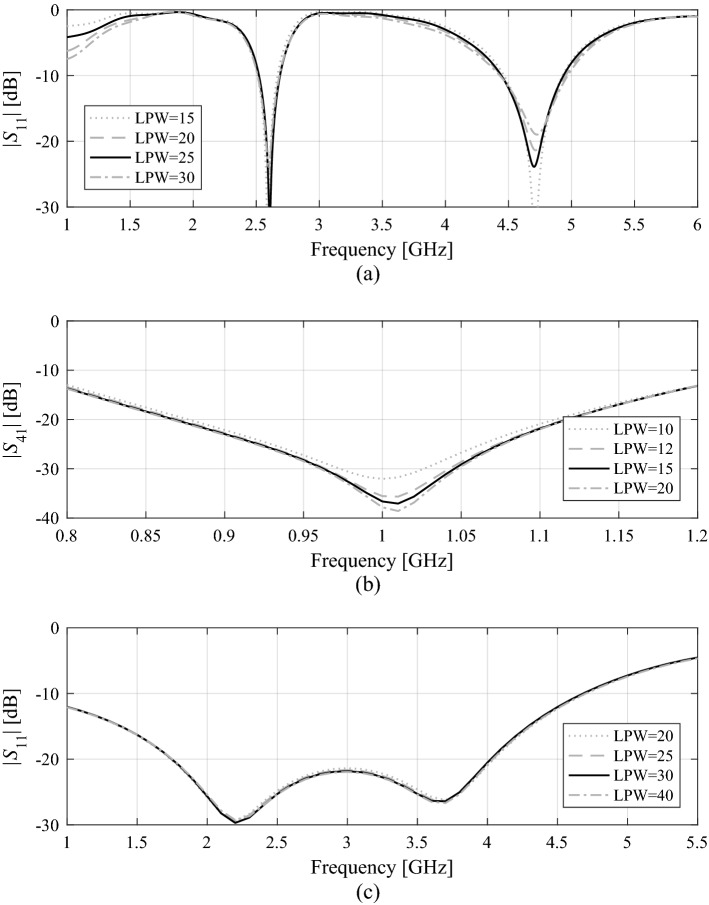
Grid convergence plots for the most representative responses of: (**a**) Structure I, (**b**) Structure II, and (**c**) Structure III for various values of LPW (lines per wavelength) parameter controlling the mesh density in CST Microwave Studio. Black lines pertain to the LPW values used in numerical experiments: 25, 15, and 30 for Structures I through III, respectively.

The experimental validation of the high-frequency structures utilized as verification case studies has been provided in their respective source papers^63–65^. Moreover, Structure I and II have been experimentally verified in our previous work, e.g.,^66,67^. Thus, the experimental validation has not been provided as being immaterial to the scope of the paper. For additional verification, Fig. 7 presents the families of the responses of Structures I through III corresponding to various model fidelities. In our work, the model fidelity is set using the parameter LPW (lines per wavelength) controlling the mesh density in CST Microwave Studio, which is utilized for performing the EM simulations.

Table 1 shows the details concerning all structures, including material parameters of the dielectric substrate, designable parameters, as well as performance specifications. The objective functions are determined in a similar way as delineated in “Identification of promising regions by inverse regression models”. In particular, the objective function for Structure I is defined according to (2) with *F*_1_ = 2.5 GHz, and *F*_2_ = 4.8 GHz. The merit function for Structure II follows (3) with *F*_1_ = 1 GHz and *F*_2_ = 0 dB. In the case of Structure III, the objective function takes the form *U*(***x***,***F***) = max{*F*_1_ ≤ *f* ≤ *F*_2_ : |*S*_11_(***x***,*f*)|}. It should be noted that the parameter spaces (last row of Table 1) are large: the average upper-to-lower bound ratio is around five, with the maximum values reaching ten for Structures I and III. The verification examples are described by six parameters (Structure I and II), and ten parameters (Structure III). In the future work, for a more comprehensive assessment of the performance of our approach, higher- and lower-dimensional examples, as well as structures characterized by multimodality will be taken into account.

**Table 1:** Verification structures.

	Case study		
	Structure I	Structure II	Structure III*
Substrate	*ε*_*r*_ = 3.5	*ε*_*r*_ = 3.5	*ε*_*r*_ = 3.5
	*h* = 0.76 mm	*h* = 0.76 mm	*h* = 0.76 mm
Design parameters	***x*** = [*l*_1_ *l*_2_ *l*_3_ *w*_1_ *w*_2_ *w*_3_]^*T*^	***x*** = [*l*_1_ *l*_2_ *l*_3_ *d w w*_1_]^*T*^	***x*** = [*l*_1.1_ *l*_1.2_ *w*_1.1_ *w*_1.2_ *w*_1.0_ *l*_2.1_ *l*_2.2_ *w*_2.1_ *w*_2.2_ *w*_2.0_]^*T*^
Other parameters	*l*_0_ = 30, *w*_0_ = 3, *s*_0_ = 0.15, *o* = 5	*d*_1_ = *d* +|*w* − *w*_1_|, *d* = 1.0, *w*_0_ = 1.7, and *l*_0_ = 15	*w*_*in*_ = 1.7, *w*_*out*_ = 0.4
Design specifications	Minimize reflection coefficient |*S*_11_| at two operating frequencies, 2.5 GHz and 4.8 GHz	Ensure 3 dB power split, |*S*_31_| −|*S*_21_|= 3 dB, at the operating frequency *f*_0_ = 1 GHz, and minimize max{|*S*_11_|,|*S*_41_|} at *f*_0_	Minimize the maximum in-band reflection coefficient |*S*_11_| within the range 1.8 GHz to 4.0 GHz
LPW	25	15	30
Simulation time^#^	60 s	160 s	120 s
Parameter space	***l*** = [15 5 5 0.2 1.5 0.5]^*T*^	***l*** = [1.0 5.0 10.0 0.2 0.5 0.2]^*T*^	***l*** = [2.0 0.1 0.5 0.1 0.2 2.0 0.1 0.1 0.1 0.2]^*T*^
	***u*** = [50 15 30 0.6 5.0 5.0]^*T*^	***u*** = [6.0 15.0 25.0 1.2 1.5 1.2]^*T*^	***u*** = [5.5 0.5 1.1 0.8 1.1 4.5 1.0 0.6 0.6 1.3]^*T*^

*Input and output line widths, *w*_*in*_ and *w*_*out*_, set to ensure 50-Ω input and 100-Ω output impedance.

^#^EM simulations have been performed on Intel Xeon 2.1 GHz machine with 64 GB RAM.

### Experimental setup and results

The three verification structures described in “Verification case studies” section have been optimized using the proposed algorithm of “Globalized search using inverse regression surrogates and response features” section, the PSO algorithm, as well as gradient-based search (benchmark methods). In all cases, ten independent runs have been executed, and the results’ statistic have been performed, in particular, the average objective function value, as well as its standard deviation as a measure of repeatability of solutions. In the case of gradient-based optimization, the search process was executed using ten random initial designs.

The setup of all algorithms was as indicated below, where *n* stands for the dimensionality of the parameter space (the meaning of the control parameters of the algorithm of “Globalized search using inverse regression surrogates and response features” section can be found in “Optimization framework” section):

- Proposed algorithm (Section Globalized search using inverse regression surrogates and response features): *N*_1_ = *N*_2_ = 2*n*, *N*_3_ = *n*, *N*_*max_global*_ = *N*_*max_local*_ = 10*n*, and *a*_*red*_ = 1.5. The threshold values *U*_*local*_ and *E*_*reduce*_ are problem dependent and set to correspond to relatively mild conditions; we set *U*_*local*_ =  − 5 dB and *E*_*reduce*_ = 0.5 (Structure I), *U*_*local*_ = 10 and *E*_*reduce*_ = 0.3 (Structure II), *U*_*local*_ =  − 15 and *E*_*reduce*_ = 0.2 (Structure III);
- PSO: swarm size *N*_*s*_ = 10, maximum number of iterations *k*_max_ = 50, weight coefficients for velocity updating: *χ* = 0.73, *c*_1_ = *c*_2_ = 2.05, cf.^62^;
- Gradient-based algorithm: trust-region framework (cf.^57^) with finite-differentiation-based sensitivity updates; termination conditions: convergence in argument ||***x***^(*i*+1)^ − ***x***^(*i*)^||< 10^−3^ or reduction of the trust region size beyond 10^−3^.

It should be noted that, despite a formally large number of the control parameters of the algorithm of “Globalized search using inverse regression surrogates and response features” section, the actual setup is simple: the number of random samples and the infill points is related to the parameter space dimensionality, whereas the threshold values are set to correspond to rather mild conditions concerning the design quality and are not critical. This is because the global search stage is only supposed to find the designs that are allocated in the region of attraction of the optimum ones (from the point of view of local search). The numerical values can be easily established by the user familiar with a given design task and the structure at hand.

For the PSO algorithm, the setup is very restrictive, which is because of high computational costs of massive EM analyses of the considered structures, about two, three and six minutes per simulation for Structures I through III, respectively. As a result, the running time of the algorithm is as high as one to two days even for the prescribed swarm size and the number of iterations. Another reason for choosing this PSO setup is to verify the quality of the solutions produced by the algorithm when its computational budget is limited to the levels comparable (although still larger) than for the proposed algorithm. The literature offers several approaches to tuning PSO algorithm to a specific design optimization case and to enhance its computational efficiency^68–71^. One of the available methods involves scaling the size of the swarm and the number of iterations of the PSO optimizer based on the number of design variables of a design optimization task at hand^68^. Due to the extremely high-cost of executing numerical experiments with the use of EM simulation tools, the analysis of the setup of the parameters of the PSO algorithm has not been conducted. This will be a guide for the future work.

Gradient-based search is used as a benchmark method in order to demonstrate that the considered design tasks are, in fact, multimodal. Therefore, it has been executed ten times from random initial design. For some of the runs, the local optima found are away from satisfactory designs, which means that the operating frequencies are not aligned with the assumed targets. For such runs, the algorithm outcome is marked as unsuccessful. The overall success rate is quantified as the relative number of runs, for which the mentioned alignment has been achieved.

**Figure 8:**
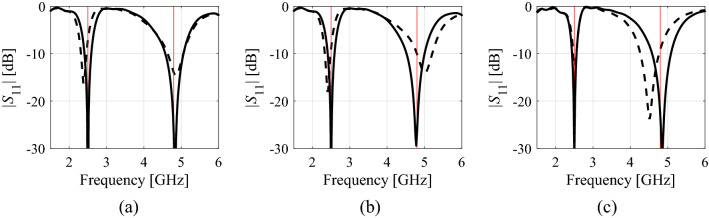
Structure I responses obtained in selected runs of the proposed algorithm: (**a**) run 1, (**b**) run 2, (**c**) run 3: design found after the global search stage (- - -), final design (—). Vertical lines mark the target operating frequencies, here 2,45 GHz and 5,3 GHz.

**Figure 9:**
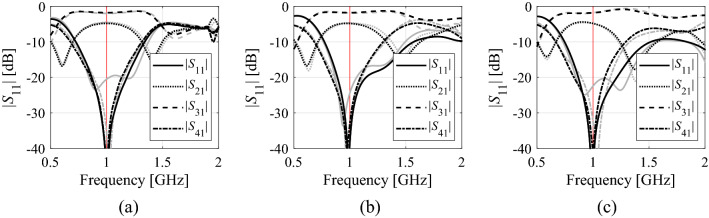
Structure II responses obtained in selected runs of the proposed algorithm: (**a**) run 1, (**b**) run 2, (**c**) run 3: design found after the global search stage (gray), final design (black). The vertical line marks the target operating frequency of 1 GHz.

**Figure 10:**
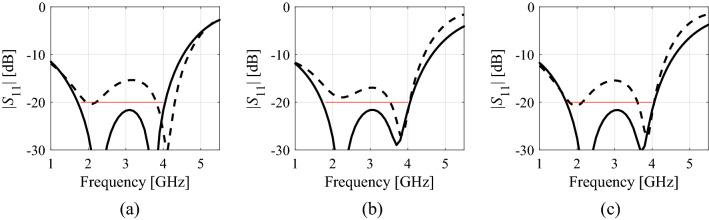
Structure III responses obtained in selected runs of the proposed algorithm: (**a**) run 1, (**b**) run 2, (**c**) run 3: design found after the global search stage (- - -), final design (—). The horizontal line marks the intended operating bandwidth, here, from 1.8 GHz and 4.0 GHz.

The numerical results have been gathered in Tables 2, 3 and 4. The data therein includes the average objective function value, the computational cost of the optimization process calculated as the number of EM simulations of the structure at hand, as well as the success rate, i.e., the number of runs for which the algorithm found the design at which the operating parameters are sufficiently close to the target. Figures 8, 9 and 10 illustrate the responses of the considered structures obtained during the selected runs of the proposed algorithm.

**Table 2:** Optimization results for Structure I.

Optimization method	Algorithm of Section “Globalized search using inverse regression surrogates and response features” (this work)	PSO		Trust-region gradient-based algorithm
		20 iterations	50 iterations	
Average objective function value (dB)	− 20.7	− 14.8	− 21.9	− 12.7
Computational cost^$^	73.3	200	500	63.8
Success rate^#^	10/10	8/10	8/10	6/10

^$^The cost calculated as the number of EM simulations of the structure under design, averaged over ten algorithm runs.

^#^Number of algorithms runs with the operating frequency allocated with sufficient accuracy, i.e., to satisfy *U*(x*,***F***) ≤ *U*_*local*_.

**Table 3:** Optimization results for Structure II.

Optimization method	Algorithm of Section "Globalized search using inverse regression surrogates and response features" (this work)	PSO		Trust-region gradient-based algorithm
		20 iterations	50 iterations	
Average objective function value (dB)	− 36.1	− 12.4	− 25.8	− 16.5
Computational cost^$^	59.6	200	500	75.4
Success rate^#^	10/10	6/10	9/10	5/10

^$^The cost calculated as the number of EM simulations of the structure under design, averaged over ten algorithm runs.

^#^Number of algorithms runs with the operating frequency allocated with sufficient accuracy, i.e., to satisfy *U*(x*,***F***) ≤ *U*_*local*_.

**Table 4:** Optimization results for Structure III.

Optimization method	Algorithm of Section "Globalized search using inverse regression surrogates and response features" (this work)	PSO		Trust-region gradient-based algorithm
		20 iterations	50 iterations	
Average objective function value (dB)	− 21.3	− 19.9	− 20.7	− 21.5
Computational cost^$^	63.2	200	500	85.8
Success rate^#^	10/10	10/10	10/10	10/10

^$^The cost calculated as the number of EM simulations of the structure under design, averaged over ten algorithm runs.

^#^Number of algorithms runs with the operating frequency allocated with sufficient accuracy, i.e., to satisfy *U*(x*,***F***) ≤ *U*_*local*_.

### Discussion

The results presented in “Experimental setup and results” section allow us to draw several conclusions pertaining to the performance of the proposed optimization framework, including both its computational efficiency and reliability. These can be summarized as follows:

- The considered problems, except for Structure III, are indeed multimodal. It has been verified that the outcome of gradient-based optimization depends on the initial design, and the success rate is only sixty percent for Structure I and fifty percent for Structure II;
- Globalized search capability has been corroborated by 100-percent success rate (in terms of identifying the design that satisfies the performance specifications) for all the considered structures;
- The quality of designs rendered by the proposed procedure is competitive over the benchmark. In particular, the average objective function values are comparable or better than for PSO, and better than the gradient-based search (except Structure III, which turns out not to be a multimodal problem).
- The computational cost of our algorithm is comparable to that of gradient-based search, which is remarkable given the problem complexity and added global search capability. As a matter of fact, the average cost is even slightly lower for Structures II and III. The reason for excellent computational efficiency is that the global search stage is capable of yielding good initial designs as indicated in Figs. 8 through 10. Consequently, subsequent local tuning can be concluded in a smaller number of iterations than the local search carried out from a random initial design. The running time is further reduced by utilization of the Broyden update in the later stages of the process, rather than full finite differentiation, FD (recall that the cost of FD-based iterations is at least *n* + 1 EM analyses, with *n* being the parameter space dimensionality).
- Based on the results obtained for the PSO algorithm, the computational budget required to surpass the design quality obtained by the proposed algorithm can be estimated as being between 500 (for Structure I) and at least 1,000 objective function evaluations (for Structures II and III). This means that the computational cost of the proposed procedure is about an order of magnitude lower than the cost of nature-inspired population-based procedures.
- In order to further investigate the performance of PSO algorithm, for each verification structure, a single optimization run has been executed with the maximum number of iterations set to 200. The obtained objective function values have been equal to − 20.4 dB, − 33.6 dB and − 21.0 dB for Structures I through III, whereas the computational cost of each run has been equal to 2000 EM simulations, which corresponds to the simulation time equal to 33 h, 88 h, and 66 h for Structures I through III, respectively. Note that the obtained objective function values are only better than for PSO executed with 500 function evaluations for Structure II and III. The reasons is poor repeatability of solutions of the PSO algorithm, meaning, that the results of individual runs may readily be worse than the average performance (evaluated over the number of runs).

At this point, one needs to reiterate that the excellent performance of the presented optimization framework comes with some limitations. In particular, the underlying assumption is that the response features are identifiable in the EM-simulated responses of the considered structure (e.g., resonances, bandwidths), at least for the designs that are of decent quality. This is generally the case for structures such as narrow- or multi-band antennas, microwave couplers, power dividers, and so on. Broadband and ultra-broadband components may be trickier, yet the definition and extraction of the response features is normally realized on case-to-case basis. On the other hand, the specific verification devices considered in this work feature distinct characteristics that are representative to a range of high-frequency passive components. This indicates a relative wide spectrum of possible applications of the proposed methodology.

## Conclusion

This paper presented a computationally efficient approach to globalized optimization of electromagnetic computational models. Our methodology involves inverse regression models constructed at the level of characteristics points of the system responses. The latter are extracted to estimate the actual operating parameters of the structure at hand, and—upon being embedded into the inverse model—guide the optimization process towards more promising regions of the parameter space. The global search stage is followed by gradient-based parameter tuning accelerated by means of sparse sensitivity updates, carried out using a rank-one Broyden formula. The proposed technique capitalizes on weakly nonlinear relationship between the operating parameters (e.g., the resonant frequencies of an antenna) and geometry variables, as opposed to highly-nonlinear responses when considered in their entirety, i.e., frequency characteristics. Further efficacy improvements are due to a normally small number of operating conditions as compared to the dimensionality of the parameter space, which is explored by the employment of inverse models. The proposed algorithm has been comprehensively validated using three high-frequency structures, a dual-band antenna, a compact microstrip coupler, and a miniaturized impedance matching transformer. The results demonstrate global search capability with a remarkably low computational cost of a few dozens of EM analyses, which is comparable to the cost of gradient-based local optimization. The technique discussed in this paper can be useful for a rapid parameter tuning of high-frequency circuits in situations where globalized search is necessary (e.g., due to the lack of reasonably good initial design), whereas computational budget is severely limited. A limitation of the method is that response features being a foundation of the inverse model, have to be extracted from EM-simulation results, which requires problem-specific routines. The future work will be focused on generalization and automation of this step of the procedure.

## Data Availability

The datasets generated during and/or analysed during the current study are available from the corresponding author on reasonable request.

## List of symbols

***A***: Regression matrix
*a*_*red*_: Multiplication factor for sampling region reduction
*β*: Penalty coefficients of the objective function *U*
*c*_1_ = *c*_2_ = 2.05: Control parameters of the PSO optimization algorithm
***d***^(^^*i*^^)^: Size vector of the TR algorithm
*E*(***p***): Merit function
*E*^(^^*j*^^)^: Merit function for particular feature vector ***p***(***x***_*r*_^(^^*j*^^)^); *E*^(^^*j*^^)^ = *E*(***p***(***x***_*r*_^(^^*j*^^)^))
*E*_*reduce*_: Threshold value for *E*(***p***(***x***_*best*_)) to reduce the sampling region (infill sampling)
EM: Electromagnetic
*f*: Frequency
*F*: Objective space
***F*** = [*F*_1_ … *F*_*N*_]^*T*^: Target objective vector
***F***^*^: Target objective vector specific to the particular design task
*F*_*T*_: Space of auxiliary objective vectors
***F***_*T*_: Auxiliary objective vector
***F***_*T*_^*^: Particular auxiliary vector corresponding to the target objective vector ***F***^*^
*f*_0_, *f*_0.1_, *…*_,_*f*_0.__*N*_: Circuit operating frequencies
FD: Finite differentiation
***G***^(^^*i*^^)^: Linear model locally approximating ***R***(***x***) at ***x***^(^^*i*^^)^
***J***_*R*_: Sensitivity matrix of the system responses
*K*: Power split
*k*_max_: Maximum number of iterations, *k*_max_ = 50 (PSO optimization algorithm)
***l***: Lower bound on design variables
*l*_*k*_, *k* = 1, …, *N*_*T*_: Level coordinates of the response features
***L***(***s***(***p***)): Inverse regression surrogate
LHS: Latin hypercube sampling
*n*: Number of design variables
*N*_1_: Number of initial random samples ***x***_*r*_^(^^*j*^^)^
*N*_2_: Number of best samples selected to construct the inverse surrogate
*N*_3_: Number of infill samples added to the sample pool per iteration
*N*_*max_global*_: Maximum number of EM evaluations for the global search stage
*N*_*max_local*_: Maximum number of EM evaluations for the local search state
*N*_*s*_: Swarm size, *N*_*s*_ = 10 (PSO optimization algorithm)
***p***(***x***) = [*p*_1_(***x***) … *p*_*L*_(***x***)]^*T*^: Vector of response features at ***x***
PSO: Particle swarm optimization
***R***(***x***): Response of the circuit EM-simulation model
*s*_*k*_(***p***), *k* = 1, …, *N*_*T*_: Scalar functions mapping the features into *F*_*T*_
***s***(***p***) = [*s*_1_(***p***) … *s*_*NT*_(***p***)]^*T*^: Vector of scalar functions *s*_*k*_(***p***)
*S*_*kl*_(***x***,*f*): Circuit *S*-parameters, *k* and *l* denote circuit ports
TR: Trust region
***u***: Upper bound on design variables
*U*(***x***,***F***): Objective function
***x***^*^(***F***) = ***x***^*^: Optimal solution
*U*_*local*_: Threshold value for *U*(***x***_*best*_,***F***^***^) to enter the local search stage
***W***: Weight matrix
***x***: Vector of designable (typically, geometry) parameters
***x***^*^: Optimal solution
***x***^(^^*i*^^)^, *i* = 0, 1, …: Approximations of the solution ***x***^*^ (TR algorithm)
***x***_*best*_: Best design found so far in the optimization process
***x***_*r*_^(^^*j*^^)^ = [*x*_*r*__.1_^(^^*j*^^)^ … *x*_*r*__.__*n*_^(^^*j*^^)^]^*T*^, *j* = 1, …, *N*_1_: Set of random points in *X*
***x***_*T*_^*^: Design rendered by inverse model used as a starting point for local optimization
*X*: Design space
*χ*: Control parameter of PSO optimization algorithm , *χ* = 0.73
